# AAPS Workshop Report on ICH M10

**DOI:** 10.1208/s12248-019-0398-7

**Published:** 2019-12-10

**Authors:** Brian Booth, Faye Vazvaei, Eric Fluhler, Heather Myler, Eric Woolf

**Affiliations:** 1 Office of Translational Sciences, Office of Clinical Pharmacology, CDER, US FDA, Silver Spring, Maryland USA; 2 Department of Pharmacokinetics, Pharmacodynamics and Drug Metabolism, Merck & Co., Inc., West Point, Pennsylvania USA; 3 BA-PK Compliance Associates, LLC, Washingtonville, New York USA; 4 Immunochemistry Department, PPD Laboratories, Richmond, Virginia, USA

## Abstract

Over the last decade, several regulatory guidelines on bioanalytical method validation (BMV) have been issued by regulatory agencies around the world. This has left the bioanalytical community struggling with regional differences in regulatory expectations when preparing for global pharmaceutical submissions. The International Council for Harmonization of Technical Requirements for Pharmaceuticals for Human Use (ICH) has the mission to achieve greater harmonization worldwide to ensure that safe, effective, and high-quality medicines are developed and registered in the most resource-efficient manner. Following calls for harmonization, ICH-selected bioanalytical method validation and sample analysis among its topics for guidance development and earlier this year released a draft guideline (M10) on BMV for public consultation. In response, the American Association of Pharmaceutical Scientists (AAPS) held a 3-day workshop to provide a forum for regulatory, industry, and academic scientists to discuss the guideline and hear various points of view on key aspects. While there was agreement that the draft guideline is generally well written and comprehensive, specific topics generated considerable discussion and, in some cases, revision recommendations for consideration by the expert working group (EWG) responsible for the guideline content. This report provides a summary of the workshop proceedings.

## INTRODUCTION

In February 2019, the International Council for Harmonization of Technical Requirements for Pharmaceuticals for Human Use (ICH) released a draft harmonized guideline (M10) on bioanalytical method validation (BMV) for public consultation. In its continuing efforts to support excellence in the pharmaceutical sciences, the American Association of Pharmaceutical Scientists (AAPS), in collaboration with the European Bioanalysis Forum (EBF), Japan Bioanalysis Forum (JBF), and China Bioanalysis Forum (CBF), organized a workshop for stakeholders from industry, academia, and health authorities to discuss and provide collective feedback on the draft guideline. This was the second in a series of sister workshops organized by the previously mentioned regional bioanalytical groups. The first forum in this series, organized by EBF, was held in May 2019 in Barcelona, Spain.

This article provides a summary of the workshop and the sections of the draft guideline that stimulated the most discussion. The discussions and proposed changes described in this manuscript summarize the views of the AAPS members in attendance and do not necessarily reflect the views or policies of the FDA or ICH M10 EWG or the authors. Furthermore, the proposed changes do not necessarily indicate that consensus was reached by all workshop participants but do provide substrate for consideration by the ICH EWG responsible for the guidance.

## BACKGROUND

It has long been recognized that bioanalysis plays an important, even critical, role in drug development and the regulatory approval process. Aiming to ensure that the best scientific practices are embraced, the bioanalytical community (industry, academic, and health authority scientists) has been actively engaged in discussions of best practices over the years. In the USA, this dialogue has, to a large extent, taken the form of multiday workshops. AAPS and the FDA have co-sponsored several of these workshops (commonly referred to as the Crystal City Meetings), the outputs of which were published (1–5) and served as substrate for consideration in development and revision of FDA regulatory guidance. (6,7) While the 2001 FDA guidance on BMV served as an industry benchmark for chromatographic assays for several years, numerous health authorities have since published their own guidelines (e.g., ANVISA 2003/2012, EMA in 2011, MHLW in 2013/2014, and the China Pharmacopeia in 2015) (8–13), in some cases including expectations for both chromatographic and ligand-binding assays. Although these guidance/guidelines share many of the same basic principles and requirements, important differences do exist. As a result, sponsors must consider the implications of these differences on global submissions, often resulting in additional work. This has led to a general call for harmonization of the various BMV guidance/guidelines (14), and in 2016, ICH announced it had endorsed “BMV and sample analysis” as a topic for development of a new guideline (M10). Following endorsement, an expert working group (EWG) with representatives from ICH member health authorities and industry organizations (e.g., PhRMA, EFPIA) was assembled and tasked with authoring the draft ICH M10 BMV guideline. The draft guideline was posted for public consultation in February 2019. In keeping with its tradition of supporting the bioanalytical community, AAPS organized a workshop, held in June 2019, to discuss the guideline, allow participants to hear both industry and health authority perspectives, and ultimately, provide collective feedback to the EWG. The meeting also offered the opportunity for participants to hear a summary of a similar “sister” meeting held by the EBF and comments provided by CBF and JBF leadership/representatives. The following sections summarize the deliberations which took place during each day of the workshop, with emphasis on topics that generated the most discussion.

## DAY ONE/SESSION ONE

This session included an introduction to the workshop and the historical landscape leading up to the M10 draft. The session also dealt with the objective, background, and scope of the guideline and the section on method development.

### Scope

The objective and background sections of the M10 draft provide the regulatory rationale for BMV and created relatively little discussion at the workshop. In contrast, the scope section of the M10 draft generated robust discussion. The scope section of M10 indicates that “This guideline describes the method validation that is expected for bioanalytical assays that are submitted to support regulatory submissions. The guideline is applicable to the validation of bioanalytical methods used to measure concentrations of chemical and biological drug(s) and their metabolite(s) in biological samples (e.g., blood, plasma, serum, other body fluids or tissues) obtained in pivotal nonclinical PK/TK studies that are used to make regulatory decisions and all phases of clinical trials in regulatory submissions.” Many workshop participants expressed concern around the use of the word “pivotal” in the document, indicating that it is too vague and open to interpretation. It was suggested that the guideline should focus on studies that are used for approval decision-making, including GLP nonclinical studies, and that the guideline needs to better describe the nonclinical PK studies that would be in scope. A proposed rewording of this paragraph that was discussed at the meeting follows:

“This guideline describes the method validation that is expected for bioanalytical assays that are submitted to support regulatory submissions. The guideline is applicable to the validation of bioanalytical methods used to measure concentrations of chemical and biological drug(s) and their metabolite(s) in biological samples (e.g., blood, plasma, serum, other body fluids, or tissue) obtained in pivotal nonclinical TK/PK *studies falling under the scope of the GLPs that are* used to make regulatory decisions, *nonclinical PK studies that are conducted as surrogates for clinical studies when no human efficacy trials can be conducted*, and all phases of clinical trials in regulatory submissions *for which a primary objective of the study is to assess, compare or characterize drug exposure*.”

The M10 draft scope also indicates that “Full method validation is expected for the primary matrix(ces) intended to support regulatory submissions. Additional matrices should be partially validated as necessary.” Workshop participants questioned whether “full” validation is possible with nonliquid matrix(ces) considering factors such as spike vs sample recovery can bias results for these matrices. There was also concern that some health authorities might refuse to accept studies using less than “fully validated” methods that fall into the ambiguous points of the scope. Workshop participants felt this latter issue could potentially be addressed with “officially issued educational material” (e.g., Q&A Document).

Finally, the scope section of M10 specifically indicates that biomarker and immunogenicity assays are out of scope of the guideline. Workshop participants proposed that *in vitro* assays should also be added as specifically out of scope.

### Method Development

The M10 draft guideline contains a relatively large section on bioanalytical method development (MD). Participants discussed that historically MD has not been within the scope of bioanalytical method validation activities and that MD data has generally not been subject to regulatory review. The presence and length of the section raised concern that MD data would now be subject to inspection and that all the MD elements listed in the guideline might be required by some agencies. In general, the need for such a detailed section was questioned.

The M10 draft states “The purpose of bioanalytical method development is to define the design, operating conditions, limitations and suitability of the method for its intended purpose and to ensure that the method is optimized for validation.” There was extensive discussion at the meeting regarding the term “optimized.” Concern was expressed that some regulatory authorities would require MD data demonstrating that each of the MD elements listed have been optimized. It was suggested that “…optimized for validation” be replaced with “…ready for validation” and that the use of the terms “optimizing,” “optimization,” and “optimized” be deleted in subsequent paragraphs of the section.

The MD section also states that “Before the development of a bioanalytical method, the applicant should understand the analyte of interest (e.g., the physicochemical properties of the drug, *in vitro* and *in vivo* metabolism and protein binding) and consider aspects of any prior analytical methods that may be applicable.” Participants indicated that method development is often conducted in some matrices before detailed information on the metabolism and protein binding of the compound are known (especially *in vivo* metabolism). Ultimately it was recommended that this sentence be deleted, since it could result in expectations that all this data be available prior to MD.

While the M10 draft indicates that MD does not require extensive record keeping, a point which was applauded by the participants, the use of good documentation practices during MD was endorsed.

Finally, the MD section indicates “… the applicant should record the changes to procedures, as well as any issues and their resolutions to provide a rationale for any changes made to validated methods immediately prior to or in the course of analyzing study samples for pivotal studies.” Discussion on this sentence included the suggestion that regulators are not looking for formal documentation and oversight of MD but rather the changes that occur after first validation. Furthermore, that health authorities want to capture the life cycle of a method and paint a clear picture of the evolutionary process the method has undergone. It was recommended that the sentence be clarified to indicate that changes made *after* initial validation are what is in scope.

## DAY ONE/SESSION TWO

### Full Validation

Session 2 in the afternoon of day 1, included full, partial, and cross validation for both chromatographic and LBA methodologies.

#### Full Validation Chromatography

##### Selectivity

The participants requested that the EWG provide more guidance in the following paragraph in case one or more of the lots tested fail: “Responses detected and attributable to interfering components should not be more than 20% of the analyte response at the LLOQ and not more than 5% of the IS response in the LLOQ sample for each matrix in 80% of the lots tested.”

##### Matrix Effect

It was acknowledged that the requirement for matrix factor determination is no longer in M10. Instead, M10 draft states, “The matrix effect should be evaluated by analyzing at least 3 replicates of low and high QCs, each prepared using matrix from at least 6 different sources/lots.” The approach was endorsed, but the participants indicated that the use of singlet analysis of each lot would be enough for this determination and proposed the following verbiage instead: “The matrix effect should be evaluated by analyzing at least 3 replicates of low and high QCs, each prepared *in singlet*, using matrix from at least 6 different sources/lots.” In addition, like the requirement for selectivity, the participants asked for additional guidance on how to proceed if one or more of the 6 sources/lots fail. Consensus was not reached on a recommended suggestion regarding this point.

##### Calibration Curve and Range

The M10 draft recommends that “The selection of the regression model should be directed by written procedures.” The participants felt that this requirement is overly burdensome, and a standard operating procedure is not needed for the selection of the model. The consensus was to remove this sentence from the guideline. There was also some confusion around the rejection of a calibrator when 50% of replicates meet acceptance criteria in the following statement: “In the case that replicates are used, the criteria (within ± 15% or ± 20% for LLOQ) should also be fulfilled for at least 50% of the calibration standards tested per concentration level. In the case that a calibration standard does not comply with these criteria, this calibration standard sample should be rejected, and the calibration curve without this calibration standard should be re-evaluated, including regression analysis.” It was proposed that this statement be either deleted or that further clarity be provided.

##### Preparation of QC’s and Evaluation of A&P

The placement of the mid-QC sample has been a subject of discussion in the bioanalytical community. The debate centers around whether the mid-QC should be placed around the geometric mean or the arithmetic mean of the calibration curve range. Although there was a consensus that this should not matter if the calibration curve is validated, the majority thought that it is more practical to place it at the geometric mean, and it was recommended that the LBA language be used for defining mid-QC: “…around the geometric mean of the calibration curve range (medium QC) and….”

The draft M10 recommends that “Within-run accuracy and precision data should be reported for each run. If the within-run accuracy or precision criteria are not met in all runs, an overall estimate of within-run accuracy and precision for each QC level should be calculated.” It is agreed that within-run accuracy and precision data should be reported for each run, and between-run precision and accuracy should be calculated by combining the data from all runs. However, participants were unclear on the application of “overall estimate of with-in run accuracy and precision,” and requested confirmation if this line meant that all individual QC results at each level, whether or not they meet acceptance, should be included in the calculation requested.

##### Carryover

Carryover is discussed under full validation, as well as under analytical run. However, the details of how it needs to be assessed during the validation exercise are not provided. It was proposed that during validation, carryover should be assessed by placing one blank sample after ULOQ in 3 A&P runs.

#### Full Validation LBA

##### Reference Standard

The draft M10 states that “It is recommended that the manufacturing batch of the reference standard used for the preparation of calibration standards and QCs is derived from the same batch of drug substance as that used for dosing in the nonclinical and clinical studies whenever possible.” The participants felt it is not practical or always possible to use the reference standard that is “derived from” the same batch and, therefore, request that the latter be replaced with “*comparable to*.”

##### Critical Reagents

The draft M10 addresses the life cycle of critical reagents: “A critical reagent lifecycle management procedure is necessary to ensure consistency between the original and new batches of critical reagents. Reagent performance should be evaluated using the bioanalytical assay.” This approach for critical reagent life cycle management is a positive addition to M10 and was applauded by the participants.

##### Specificity

The draft M10 recommends that the ULOQ should also be investigated for evaluation of specificity: “The accuracy of the target analyte at the LLOQ and at the ULOQ should be investigated in the presence of related molecules at the maximal concentration(s) anticipated in study samples.” The participants requested that the requirements for this evaluation *at the ULOQ* be deleted as it adds little to no value.

### Cross Validation

There was quite a bit of deliberation around the M10 recommendations for cross validation. The cross validation approach that the draft M10 has proposed is new to the bioanalytical community. It was deemed important to include some context as to why cross validation is needed. The participants proposed the following statement for inclusion in M10: “Cross validation is conducted to evaluate the bias between methods (or laboratories) such that the results from studies using them can be appropriately interpreted. Cross validation allows the comparison of two methods (labs) and informs us how they are related.” Overall, the participants were open to the recommended approach and agreed to embrace it with some caveats, in particular, pointing to the need for additional clarity around what “different fully validated methods” in the following statement mean: “Data are obtained from different fully validated methods across studies that are going to be combined or compared to support special dosing regimens, or regulatory decisions regarding safety, efficacy and labelling.” In addition, it was acknowledged that in this approach, cross validation can be viewed as a characterization exercise and therefore does not have an acceptance criterion. Although this concept is new to the BA community, it is an acceptable proposition. Moreover, the participants stated that there needs to be systematic education within the industry over who owns the decision/impact/application as to how and when a correction factor is needed and applied? Additional considerations are, the generation of secondary results on clinical samples, complications associated with the patient’s informed consent, and sample export issues for limitations on shipping incurred samples out of China. Therefore, the participants requested changing the current language to allow the assessment by measuring QCs and/or study samples. Finally, given the reasoning behind the need for cross validation, the participants asked to exclude nonclinical samples.

### Partial Validation

Partial validation remains subjective as there are many different scenarios wherein a partial validation may be sufficient. With that said, a partial validation can range from one analytical run to almost a full validation leaving out repetition of the stock stability. Examples discussed were “change from one matrix within a species to another (e.g., switching from human plasma to serum or cerebrospinal fluid) or changes to the species within the matrix (e.g., switching from rat plasma to mouse plasma).” Also, the risk of transferring stability data between CRO/Pharma was discussed. Many companies/CROs may consider stability data owned by another company as not shareable and, therefore, would generate additional work.

#### Reagent Qualification

The audience thought that the use of “retest date” is a positive addition in M10. Further clarity was requested in the definition of minor vs major changes to critical reagents. The participants proposed defining “major” as non-bridgeable and “minor” as bridgeable as demonstrated in one run. Further, it was proposed by the participants that reagent qualification be documented in a Certificate of Analysis (CoA), Certificate of Testing (CoT), lab notebook, or any traceable documentation system.

## DAY TWO/SESSION ONE

Day two of the workshop was kicked off with a summary of the consensus from day one. Immediately after, the discussions started, beginning with the topic of stability, followed by sample analysis and analytical run.

### Stability

As anticipated, given the many arguments that have accumulated over numerous discussions in recent years, the topic of co-administered drug matrix stability generated a significant amount of dialogue and discussion at the M10 workshop. The draft M10 calls out the need for evaluating the stability of co-administered compounds in the following statement: “If multiple analytes are present in the study samples (e.g., studies with a fixed combination, or due to a specific drug regimen) the stability test of an analyte in matrix should be conducted with the matrix containing all of the analytes.” The workshop participants were unable to present any examples of a co-administered compound impacting the stability of another in biological fluids. The argument was made that the potential of nano-molar concentrations of a co-administered drug impacting the stability of another in a highly complex matrix is unlikely, and, therefore, the justification for efforts associated with this exercise is unclear.

Moreover, in a survey conducted by the Global CRO Consortium (GCC), there were no examples of stability issues caused by the co-administration of drugs reported in stability experiments from 28 CROs across 56 different combinations of co-administered xenobiotics (15). Although no cases could be referenced, there was acknowledgment that in some cases, the presence of one analyte might have the potential to pose a concern with the stability of another, potential scenarios included enzyme replacement therapies, inter-drug reactivity, or cases where the pH of the matrix could be impacted by the presence one drug.

The current draft lists the need for this requirement in “Method Development Section”. Given the complexity of this issue and the lack of examples of impact on stability in biological fluids, the participants felt that the co-medication stability should be independent of modality and presented in a separate section on its own. Further, in alignment with the above considerations, it is proposed that a tiered approach should be allowed for this stability beginning with a paper argument *in lieu* of actual matrix stability measurement. For co-formulated combinations, it was suggested that *in vitro* or CMC data could be used as a surrogate to support matrix stability. And finally, if needed, provide bench top assessment before a long-term stability measurement is performed. It is worth noting that there is already precedence for this approach based on a redacted Clarifax communication between Health Canada and a sponsor in which the Clarifax allows the following action: “If well-designed matrix stability are not available, [Division of Biopharmaceutics Evaluation] is willing to consider other relevant data (e.g., based on the physiochemical characteristics of [redacted[ and [redacted], the results of incurred samples reanalysis for clinical samples stored under conditions and duration supportive of those applicable to those from Studies [redacted] and [redacted].”

Finally, the workshop participants proposed that in addition to allowing for the tiered approach the language in line 403–405 stating, “[i]f multiple analytes are present in the study samples (e.g., studies with a fixed combination, or 404 due to a specific drug regimen), the stability test of an analyte in matrix should be conducted 405 with the matrix containing all of the analytes” and should be changed to the following: “For fixed dose combination and specific drug regimen where the primary objective is PK assessment, the stability test of an analyte in matrix should be conducted with the matrix containing all of the dosed compounds. ”Furthermore, the participants asked that the guideline should exclude this requirement for drug-drug interaction studies.

The other highly debated stability topic was the draft M10 requirement for the number of tubes initially imposed by Health Canada in their October 8, 2015 Notice: Clarification of bioanalytical method validation procedures (16). Although not a requirement in the FDA 2018 guidance or any other guidelines, this requirement has been worked into the draft M10: “Stability of the analyte in the studied matrix is evaluated using low and high concentration stability QCs. Aliquots of the low and high stability QCs are analysed at time zero and after the applied storage conditions that are to be evaluated. A minimum of three stability QCs should be prepared and analysed per concentration level/storage condition/timepoint.” The workshop participants felt that, to date, no evidence has been provided that the number of tubes in the storage would affect the stability outcomes. There was, therefore, a very strong proposal to remove the requirement for the number of tubes, as no guideline/guidance requires this, and there is no supporting data justifying its necessity.

The philosophy behind the need for processed sample stability remains questionable for the industry. Bioanalysis is performed in a series of experiments wherein the calibrators, quality controls, and the study samples are processed together and quantified in one analytical run. Under no circumstances should the samples be analyzed or quantified against calibrators that were processed in different analytical runs. The requirements for quantifying the stability samples against a set of calibrators prepared in a different run is confusing to the industry and may result in inappropriate bioanalytical practice. Therefore, the participants propose removing the section pertaining to “process stability” as no additional data is provided by this experiment. The M10 requirement for reinjection reproducibility should cover the need for reinjection justification.

Additional stability topics that were discussed included the bracketing approach for stability requirements for large molecules and whole blood stability. The workshop participants proposed making this requirement consistent with small molecules, wherein stability in lower temperatures can be inferred at any given state if the stability at a higher temperature is established. In addition, the participants asked to exclude the need for whole blood stability for serum.

The stability discussions were concluded on a positive note with the participants expressing appreciation for the verbiage in M10 for reagent stability indicating “the testing of the reagents should be based upon the performance in the bioanalytical assay and be based upon general guideline for reagent storage conditions and can be extended beyond the expiry date from the supplier with appropriate documentation.” However, to reflect the current industry approach, it was recommended that a change be made from “expiry” to “*expiry/retest*” in the statement above.

### Study Samples Analysis

#### Carryover

By and large, the workshop participants recognized the need for a proper assessment of carryover where applicable. Carryover is specifically applicable to chromatographic assays and generally not a concern for LBA assays; however, some LBA platforms are also prone to carryover, although the participants thought that no matter what the technology, when applicable, carry-over should properly be minimized, assessed, and any impact evaluated. To that effect, they felt that the verbiage in the FDA guidance best describes the current industry practice and recommend the carryover verbiage from lines 360 to 368 in draft M10 be replaced with, “Carryover between samples can occur in analytical methods. The sponsor should eliminate any carryover during method development. If carryover cannot be eliminated, the sponsor should assess the impact of any carryover during method validation on the accuracy of the study sample concentrations.”

#### Analytical Run Chromatography

One of the topics that produced a spirited debate without reaching a consensus was the placement of the quality control samples in an analytical run. The draft M10 recommends: “An analytical run consists of a blank sample (processed matrix sample without analyte and without IS), a zero sample (processed matrix with IS), calibration standards at a minimum of concentration levels, at least 3 levels of QCs (low, medium and high) in duplicate (or at least 5% of the number of study samples, whichever is higher) and the study samples to be analyzed. The QCs should be divided over the run in such a way that the accuracy and precision of the whole run is ensured. Study samples should always be bracketed by QCs.” The workshop participants could not reach a harmonized interpretation of the last sentence stating that the study samples should always be bracketed by QCs and recommend deleting this sentence from the guideline to allow more flexibility.

In addition, M10 is calling for dilution QCs in analytical runs, a requirement not presently in the FDA guidance. Since the precision and accuracy of the dilution is demonstrated during validation, the workshop participants asked that the following statement be deleted from M10: “Analytical runs containing samples that are diluted and reanalyzed should include dilution QCs to verify the accuracy and precision of the dilution method during study sample analysis. The concentration of the dilution QCs should exceed that of the study samples being diluted (or of the ULOQ) and they should be diluted using the same dilution factor. The within-run acceptance criteria of the dilution QC(s) will only affect the acceptance of the diluted study samples and not the outcome of the analytical run.” In addition, the wording “…samples that are diluted and reanalyzed should include dilution QCs…,” is unclear. This verbiage implies both conditions need to be met which is not the intent that “runs with diluted samples should include dilution QCs.”

The draft M10 states that “If a narrow range of analyte concentrations of the study samples is known or anticipated before the start of study sample analysis, it is recommended to either narrow the calibration curve range, adapt the concentrations of the QCs, or add new QCs at different concentration levels as appropriate, to adequately reflect the concentrations of the study samples. At the intended therapeutic dose(s), if an unanticipated clustering of study samples at one end of the calibration curve is encountered after the start of sample analysis, the analysis should be stopped and either the standard calibration range narrowed (i.e., partial validation), existing QC concentrations revised, or QCs at additional concentrations added to the original curve within the observed range before continuing with study sample analysis. It is not necessary to reanalyze samples analyzed before optimizing the calibration curve range or QC concentrations.” There was significant concern around this verbiage as it could delay dose escalation, especially considering that the range is previously validated and monitored within run using existing calibrators and QC’s; as such, the justification for this requirement was questioned as it adds additional unnecessary work.

Another discussion topic within the chromatographic analytical run included, provision of clarity in the definition of “reintegration”. It was suggested by the participants that the definition of the reintegration should be aligned with the GBC A2 whitepaper (17): Reintegration refers to changes made to integration parameters made after initial quantitation occurs. Chromatogram integration and reintegration should be described in a study plan, protocol, or SOP. Any deviation from the procedures described *a priori* should be discussed in the Bioanalytical Report. The list of chromatograms that required reintegration, including any manual integrations, and the reasons for reintegration should be included in the Bioanalytical Report. Original and reintegrated chromatograms and initial and repeat integration results should be kept for future reference and submitted in the Bioanalytical Report for comparative BA/BE studies.”

Finally, for multi-analyte assays, although M10 allows independent assessment of each analyte, it nevertheless calls for re-extraction of the failed analyte. The participants argued that “reinjection” of the analytical run should also be allowed as in many cases, the failure could be due to such instrument issues as drift.

#### Analytical Run, Both LBA and Chromatography

One of the most critical statements in M10 is with respect to the overall study performance statistics for QC samples. The guideline advises that if the “overall mean accuracy or precision fails the 15% criterion,” the sponsors should investigate the reasons for this high variability. The guideline further warns the applicant, that in the case of comparative BA/BE studies, a high variability in performance QCs may result in the rejection of the data. The participants have asked for the removal of this verbiage as all samples are reported from independently acceptable runs. From time to time, a QC sample may fall out of the acceptance criteria and may influence the overall precision and accuracy without an identifiable cause. This requirement for investigation and the potential rejection of the study has no scientific merit.

The participants commented that throughout the technical document, the words “batch,” “run,” or “plate” were sometimes used interchangeably, making it difficult for the readers. In addition, the use of the statement “multiple batches” within one analytical run lacks clarity for LBA methods, as this is not a common practice. The group suggested clarifying these terms and aligning the verbiage across both chromatographic and LBA sections as applicable.

#### Analytical Run LBA

There was a positive notation that generally, this section was consistent with the current industry practice. The concept of “single-well” determinations for LBA was applauded and welcomed by the industry.

## DAY TWO/SESSION TWO

This session dealt with the topics of reanalysis (including ISR), analytes that are also endogenous, diagnostic kits, method validation to support new modalities, and documentation.

### Reanalysis

The M10 draft lists a number of examples where the need for study sample reanalysis is recognized. These include rejection of the analytical run, inappropriate dilution, and quantifiable levels in pre-dose samples. Additionally, the M10 draft indicates that for comparative bioavailability/bioequivalence studies, reanalysis for PK reasons is not acceptable. There was little to no discussion at the workshop on the majority of these examples, as they are generally currently recognized by the bioanalytical community.

The one example pertaining to sample reanalysis that did generate discussion related to samples analyzed in multiple replicates, as is currently the case for the majority of ligand-binding assays. The current M10 draft states that reanalysis is required “when samples are analyzed in more than one well and non-reportable values are obtained due to one replicate failing the pre-define acceptance criteria (e.g., excessive variability between wells, one replicate being above the ULOQ or below the LLOQ).” Discussion at the workshop centered around the point generally; the back calculated concentrations of individual wells are not calculated; rather instrument response is the measurement assessed for determination of well-to-well variability. This being the case, workshop participants recommended deletion of the verbiage pertaining to one replicate being above the ULOQ or below the LLOQ.

In addition to specifying appropriate rationale for reanalysis, the M10 draft calls for a listing of reanalyzed samples in the bioanalytical report. The listing is to include a number of parameters, including the initial analysis value. It was discussed, that in the cases where reanalysis is required due to run failure or inappropriate dilution, there is no “initial analysis value” to include in the reanalysis table, as the result from the initial analysis is not valid.

Finally, the draft guideline specifies that multiple determinations are required when the initial analysis value requires confirmation (e.g., pre-dose sample with a measurable concentration). The specification of multiple determinations was viewed as ambiguous; hence, it was recommended that the guideline state that “at a minimum, duplicate determinations are required” in such cases.

### Incurred Sample Reanalysis (ISR)

Incurred sample reanalysis has, since the Crystal City III meeting in 2006, been a topic of active discussion within the bioanalytical community. ISR is a component of the current bioanalytical method validation guidance/guideline from both the US FDA and the EMA. The M10 draft lists a number of specific study types for which ISR is required. These include all pivotal comparative BA/BE studies, the first clinical trial in subjects, pivotal early patient trials (once per patient population), as well as the first trial in patients with impaired hepatic or renal function. With respect to nonclinical studies, the draft states that “ISR should, in general, be performed for the main nonclinical TK studies, once per species.” Inclusion of an explicit list of studies for which ISR is required was viewed positively by workshop participants. In fact, discussion emphasized that performing ISR in studies other than those specified would potentially lead to regulatory creep.

Discussion at the workshop pertaining to ISR focused on clarification of the purpose of ISR, as well as the number of samples required for ISR assessment.

The M10 draft states that ISR “is a necessary component of bioanalytical method validation.” The text, as written is viewed to be inaccurate, as ISR requires samples from dosed study participants and hence cannot be a component of validation activities which take place in advance of sample analysis. Rather, workshop participants recommend stating that “ISR is needed to provide confidence that the validated bioanalytical method is delivering reliable data for the study samples.”

Regarding number of samples required for the ISR assessment, the M10 draft proposes requirements identical with those contained in the current EMA guideline. That is, 10% of the first 1000 samples plus 5% of the number of samples exceed 1000. Given that ISR is generally recognized by the bioanalytical community as a method to ensure reliability of the validated method as opposed to a quality check on the sample analysis process, it was proposed that language in the guideline be revised to specify boundaries on the number of samples required for ISR assessment. The proposal from the workshop was that 10% of the study samples be subjected to ISR, with a minimum of 20 and a maximum of 100 samples. Currently, the M10 draft guideline states that these ISR samples should be prepared in the same manner as the original analysis. Use of the term prepared was viewed as potentially ambiguous; hence, it is suggested that the term “analyzed” be used in place of “prepared.”

### Analytes Which Are Also Endogenous

The M10 draft contains significant language around analytes which are also endogenous. In contrast to biomarkers, which are explicitly not within scope for M10, endogenous analytes are therapeutics/drugs that are administered to patients for which bioanalysis is required to support product registration. Examples of such analytes are testosterone and vitamin D.

The draft lists several approaches that may be used to analyze such compounds. The focus of discussion at the workshop was on the limitations associated with such methods which are not viewed to be adequately outlined in the draft. Specifically, it was recommended that surrogate analyte and standard addition approaches be noted as only being applicable for methods with linear responses and hence are not appropriate for analytes quantitated with ligand-binding methods. Additionally, with respect to standards addition, it was questioned as to whether this approach was feasible for studies with large numbers of samples. Rather, it was proposed that the standard additions approach be used with pooled incurred samples to evaluate parallelism.

Additional workshop recommendations related to endogenous analytes were to remove “Other Regions Section” (parallelism), as this topic was viewed to be covered adequately both in the glossary and “Other Regions Section”. Also, it was pointed out that absolute recovery of endogenous analytes cannot be determined as the nominal endogenous analyte concentration is unknown. Rather, it was suggested that the section covering recovery be reworded to allow determination by spiked analyte recovery and complimented by appropriate parallelism determination to ensure accurate quantification of analytes in study samples against a prepared calibration curve. Finally, it was recommended that stability of surrogate analytes be assessed in addition to that of un-spiked and spiked authentic matrix, as well as surrogate matrix, if used.

### Commercial and Diagnostic Kits

“Other Regions Section” of the M10 draft deals with the “repurposing” of commercial and diagnostic kits to support activities within the scope of the guideline. The draft requires that these kits be (re)validated to the standards specified in the document. These include using a minimum of 6 nonzero calibrators, using the analyte as reference standard, specifying actual QC concentrations as opposed to a reference interval and using the sample matrix-based calibrators and quality controls. There was little discussion on this aspect of the draft guideline; it was felt that workshop participants were in general agreement with the contents of the draft.

### New Modalities and Technologies

In recognition of the fact that new modalities will be brought forward as therapeutics in the future and that new analytical approaches will be necessary to support these modalities, “Other Regions Section” of the draft guideline deals with these topics. The overall recommendation that acceptance criteria be developed *a priori* for these new technologies and that these criteria be based on method development activities and verified during assay validation was accepted without comment by workshop participants. It was also accepted that when multiple bioanalytical platforms are used to support compound development that understanding how data from the multiple platforms relate and that potential differences between methods are well understood is a regulatory expectation.

### Documentation

The M10 draft guideline contains extensive content pertaining to bioanalytical documentation. Topics discussed in the guideline include method development documentation, laboratory records, assay validation reports, sample analysis reports, and submission documents.

With respect to method development documentation, discussions at the workshop confirmed that regulators are not looking for formal documentation of method development. Also, with respect to method development, it was recognized that documentation of changes that occur after the initial validation are of interest to the regulators, in contrast to documentation of the initial method development. Regulators want to capture the life cycle of a method and see a clear picture of the evolution of the method. Additionally, regulators need to understand changes to problematic methods which have been used for sample analysis. Such information pertaining to the method should be included in marketing submissions.

Regarding bioanalytical reports, workshop participants expressed concern that the amount of documentation currently specified in the draft is excessive.

First, it was suggested that documentation requirements for bioequivalence (BE) studies be delineated separately from other study types. The thought behind this suggestion being that additional information in BE study reports may reduce the need for on-site audits, however, such additional documentation may not offer any additional value for non-BE studies.

Second, it was recommended that requirements for specific types of documentation (i.e., logs, run sheets, etc.) be removed from the guideline, as while all regulated laboratories document these items, not all laboratories maintain items named specifically as listed in the guideline.

Sample storage information requirements were viewed as particularly excessive. A high-level statement in reports regarding sample storage was viewed appropriate. Furthermore, it was discussed that granular information such as sample chain of custody is lab specific and is potentially of minimal value to include within reports. Similarly, requiring granular data regarding blank matrix lots and instrument IDs was also felt to be of minimal value, as context may be lost.

The current M10 draft specifies that 100% of chromatograms be included in bioanalytical reports for comparative bioavailability/bioequivalence studies. Given study size, this requirement was viewed as potentially adding thousands of pages to reports. Consensus of workshop participants was that the current US FDA guidance and EMA guideline calling for inclusion of 20% of chromatograms for BE studies and 5% of chromatograms for other study types was appropriate. The group agreed that additional chromatograms could be provided upon request. Furthermore, only the final integrated chromatograms upon which final quantitative results were based were recommended for inclusion in reports. Original/raw chromatograms would be available for on-site inspection.

Other documentation recommendations from the workshop include (1) elimination of the requirement for internal standard (IS) response plots, with the understanding that the process of assessing response is defined by each laboratory by SOP, and (2) elimination of the requirement for a separate study correspondence file; correspondence pertaining to study conduct should be maintained with the study records.

Finally, clarity was requested on what should be included in the documentation pertaining to inspection audit reporting; it was recommended that regulators across the globe collaborate regarding laboratory inspection histories, rather than requiring these to be included in regulatory submissions.

## OTHER REGIONS

In the concluding session of the workshop, representatives from “sister” bioanalytical organizations (EBF, CBF, and JBF) presented the outcomes of the discussions of their respective organizations on the M10 draft. Recommendations of these organizations generally mirrored those reached by workshop participants. None of the proposals of these organizations were in direct conflict with those of this workshop. In addition, several points that were only briefly touched upon during the workshop were explicitly mentioned by these groups.

Specifically, based on member feedback, the EU based BA community feels that a (global) modern and science-based guideline should consider animal welfare and not require unnecessary use of animals (i.e., 3Rs – replace, reuse, refine). As such, EBF is proposing that the final version of M10 permits/encourages the use of surrogate matrices for calibrators/dilutions, specify the use of fewer replicates for non-clinical assays, and encourage microsampling to reduce animal stress and eliminate the need for satellite groups in preclinical rodent studies.

The CBF representative emphasized the challenge of navigating the Chinese import/export regulations with respect to bioanalytical work. As such, they expressed particular concerns over mandating the use of post-dose samples for cross validation studies between laboratories inside and outside of China. During the discussion after the CBF presentation, some of the logistical challenges of conducting regulated bioanalytical work in China were discussed. Inclusion of text in M10 to supersede regional requirements, for example, the need to use geographically matched control matrix, would be viewed as a welcome addition by the bioanalytical community.

## CONCLUSIONS

The 2.5-day workshop permitted robust discussion on the majority of the concerns of industry on the M10 draft. As a result of the workshop, AAPS has submitted the proposals upon which consensus was reached to the FDA through the website that they have established to receive comments on M10. It is hoped that these will be reviewed by members of the M10 EWG and that the final version of M10 will take these into account.
